# Chemical and Antimicrobial Analyses of *Juniperus chinensis* and *Juniperus seravschanica* Essential Oils and Comparison with Their Methanolic Crude Extracts

**DOI:** 10.1155/2021/9937522

**Published:** 2021-08-30

**Authors:** Al-Dhafri S. Khamis, Lay Ching Chai

**Affiliations:** ^1^Institute of Biological Sciences, Faculty of Science, University of Malaya, Kuala Lumpur 50603, Malaysia; ^2^Department Supervisor, Department of Biology College of Science, Sultan Qaboos University, Muscat 123, PO Box 36, Oman

## Abstract

*Juniperus chinensis* and *Juniperus seravschanica* are commonly used in the traditional folk medicine to treat microbial infection. In this study, the essential oils obtained from the leaves of *J. chinensis* growing in Malaysia and *J. seravschanica* growing in Oman were analysed by head space-solid phase microextraction-gas chromatography mass spectrometry (HS-SPME-GC-MS) and screened for antimicrobial activities against *Escherichia coli* (NCTC 10418), *Pseudomonas aeruginosa* (NCTC 10662), *Bacillus subtilis ATCC6059, Micrococcus luteus* (ATCC 9341), *Staphylococcus aureus* (NCTC 6571), and methicillin-resistant *S. aureus* (MRSA; ATCC 33591). To compare the antimicrobial activities of extracts using different extraction methods, methanol extraction was performed to obtain crude extracts from the leaves of *J. chinensis* and *J. seravschanica* for antimicrobial analysis. The HS-SPME-GS-MS analysis of the essential oils from the leaves of *J. chinensis* and *J. seravschanica* identified 37 and 36 components, respectively. Essential oils from these two species had distinctive chemical component profiles, with *α*-pinene (27.2%) as the major component of *J. chinensis* essential oil, while dl-limonene (45.2%) constitutes the major component of *J. seravschanica* essential oil. Essential oils of these two species shared only six similar terpenoids compounds: *α*-pinene, *β*-pinene, *γ*-elemene, sabinene, elemol, and 3-cyclohexen-1-ol. Overall, the essential oils showed antimicrobial activities against all the six bacterial strains tested, with the highest antagonistic activity against *M. luteus* and *B. cereus*; while, methanolic crude extracts showed the highest activities against *S. aureus* and MRSA strains. The methanolic crude extracts demonstrated significantly higher antibacterial activity against the Gram-positive bacteria (*p* < 0.005); while, the essential oils of *Juniperus* did not show significant differences between Gram-positive and Gram-negative bacteria. Future studies are needed to investigate the active compounds present in the essential oils and methanolic crude extracts that confer the selectivity in the antimicrobial activity.

## 1. Introduction

Asia has a large number of medicinal plants that have been used traditionally as folk medicine. In fact, of the 70,000 species of medicinal plants used in traditional medicine globally, a huge proportion are actually originated from Asia. Of these Asian traditional plants, *Juniperus* tree is one of the most popular and extensively studied medicinal plants. *Juniperus* belongs to the Cupressaceae family consisting of approximately 70 species that are widely distributed throughout the Northern Hemisphere. Some of the most popular species includes *J. chinensis*, *J. excelsa*, and *J. communis*. Research studies of the various species of *Juniperus*tree revealed a wide range of unique medicinal properties such as antimicrobial [1], anticancer [2] antioxidant [3], antitumor [4], antidiabetic [5], antimicrobial [6], and many other therapeutic properties [7].

*Juniperus seravschanica*, which is also commonly known as Pashtun juniper, is widely distributed in the central Asia from the southern Kazakhstan through Kyrgyzstan, Tajikistan, eastern Uzbekistan, and Turkmenistan to northern and eastern Afghanistan, northern Pakistan, and Kashmir, as well as in the mountains of southeastern Iran and the Al Hajar Mountains of Oman [8]. The Pashtun juniper is used by the Himalayans to treat a range of diseases including abdominal cramps, diarrhoea, asthma, headache, gonorrhoeae, and leucorrhoea [9]. The Lebanese and Turkish use *J. seravschanica* to treat asthma, cough, common cold, bronchitis, pneumonia, throat inflammation and tuberculosis, urinary tract inflammations, rheumatism, and to remove renal and gall bladder stones [9].

On the other hand, *J. chinensis*, which is also known as the Chinese juniper, is commonly found in many regions in the east and southeast Asia including China, Taiwan, Myanmar, Japan, Korea, and Malaysia [10]. The stems of *J. chinensis* is used in the Chinese medicine to treat parasitic skin problems and rheumatism; the fruit is used in the treatment of convulsions, excessive sweating, and hepatitis; and the root is used to treat burns and scalds [11].

This study aims to determine and compare the chemical compositions of the essential oils obtained from the leaves of the Omani *J. seravschanica* and the Malaysian *J. chinensis*. The antimicrobial activity of the essential oils and methanolic crude extracts from the leaves of the Omani *J. seravschanica* and the Malaysian *J. chinensis* were also determined.

## 2. Materials and Methods

### 2.1. Plant Materials

Two types of plant species were examined in this study, namely, *Juniperus seravschanica* and *Juniperus chinensis*. *J. seravschanica* was from Oman, while *J. chinensis* was from Malaysia.

The leaves of *J. seravschanica* were collected from the tree grown in the Jabel Akhdar Mountains of northern Oman). This plant was previously thought to belong to the Persian juniper population (*J. excelsa* subsp. *polycarpos*) but has been recently confirmed as the *Zeravshan juniper J. seravschanica* [8]. It is one of the dominant species native to the montane woodlands of Oman [12]. The specimen was authenticated at the Herbarium of the Plant Unit, Biology Department, Sultan Qaboos University, Oman, with voucher code Al-Dhafri, Kh. s.n.

The leaves of *Juniperus chinensis* were collected from the trees grown in the University of Malaya campus located in Kuala Lumpur, Malaysia. The leaves of *J. chinensis* are needle shaped when young and scaly when mature [13]. For this particular plant specimen used in this study, the leaves were spiny and had a maximum diameter of 7.5 cm. The identity of the plant was confirmed by a botanist, Dr. M. Sugumaran, of the Institute of Biological Sciences, Faculty of Science, University of Malaya, Malaysia. A voucher of the specimen was deposited at the University of Malaya Herbarium with the code number KLU047364.

### 2.2. Essential Oils Extraction

Essential oil was extracted from the leaves of *J. chinensis* and *J. seravschanica* via using the modified steam distillation method developed by Masango [14]. In brief, 500 g of leaves sample was placed in the distillation flask that was connected to the steam generator and a condenser to collect the essential oil. After that, the water was heated to 100°C to produce steam to volatilize the essential oils from the leave samples. The volatilization process was conducted for about 5 hours. Then, the mixture was collected and separated using a separatory funnel, in which the essential oils that settled at the bottom layer of the separatory funnel were collected in a clean bottle for analysis.

### 2.3. Preparation of Methanol Extracts

Leaves of the respective plants (*J. chinensis* and *J. seravschanica*) were washed with sterile water and then sun-dried for 48 h. The sun-dried leaves were then grinded to fine powder with a stainless-steel blender. After that, each powdered sample (30 g) was macerated in 500 mL of 80% methanol (Sigma-Aldrich, St. Louis, MO, USA) for 3 days at 4°C to extract the phytochemicals presence in the leaf samples [3]. The liquid extract was subsequently filtered and concentrated using a rotary vacuum evaporator (BUCHI Rotavapor, Switzerland), at 40°C. The resulting semisolid extracts which had a yield of about 15% (w/w) was then freeze-dried with a freeze dryer (Flexi-Dry MP, FTS Systems, China) into the dry fine-powdered sample, which was subsequently dissolved in sterile distilled water to an extract stock solution at a concentration of 32 mg/mL. The extract stock solutions were kept at −80°C until tested.

### 2.4. Head Space-Solid Phase Microextraction-Gas Chromatography Mass Spectrometry Analysis

The essential oils extracted from *J. seravschanica* and *J. chinensis* were analysed using the head space-solid phase microextraction-gas chromatography mass spectrometry (HS-SPME-GC-MS) method. The solid phase microextraction fibre was exposed to the headspace of the extracted essential oils, and the sample fibre was then inserted to the injection port of a Shimadzu gas chromatograph model GC-MS-QP/5050A, equipped with a quadrupole mass spectrometer and a J&W Scientific DB-5MS (5% phenyl/95% dimethylpolysiloxane) fused silica capillary column (30 m × 0.25 mm I.D. x 0.25 mm film thickness). The oven temperature was programmed to increase from 51°C to 251°C at 3°C/min. Injector and interface temperatures were kept at 275°C and 300°C, respectively. Helium was used as the carrier gas with a linear velocity of 44.6 cm/s, column flow rate of 1.5 mL/min, and total flow rate of 36 ml/min. The split ratio was 1 : 21. Mass spectra were continuously recorded over the mass range 35–501 amu. The MS operating parameters were as follows: ionization voltage 70 eV and scan rate 500 amu/s. The mass spectra obtained were compared to those recorded in the computer MS library (Wiley 229,000 database). The percentage composition was determined by using the single area percentage method without considering corrections for response factors.

### 2.5. Antimicrobial Assay

Bacteria strains used in this study were revived from glycerol stock cultures maintained in the laboratory. The Gram-positive bacteria used were *Bacillus subtilis* (ATCC6059), *Staphylococcus aureus* (NCTC 6571), *Micrococcus luteus* (ATCC 9341), and methicillin-resistant *S. aureus* (ATCC 33591); while, the Gram-negative bacteria used were *Pseudomonas aeruginosa* (NCTC 10662) and *Escherichia coli* (NCTC 10418). The bacteria were subsequently subcultured on nutrient agar (BDH Media, London, United Kingdom), and the purity of each strain was determined prior to the antibacterial assay.

The antimicrobial activities of the methanolic extracts of *J. seravschanica* and *J. chinensis* and the essential oil of *J. seravschanica* and *J. chinensis* were evaluated against the six strains of bacteria. The bacterial inoculums were prepared from overnight broth culture in physiological saline (0.8% of NaCl) to reach the turbidity equivalent to 0.5 McFarland standard (∼10^8^ cfu/mL) and further diluted to 10^6^ cfu/mL. Muller–Hinton agar (MH agar; from Sigma-Aldrich, USA) were poured in Petri dishes, solidified, and surface dried before inoculation. The extracts were dissolved in deionized water to obtain a final concentration of 10 mg/mL, and the suspension was filter-sterilized using the 0.22 *μ*m membrane filter. Sterilized 6 mm filter paper discs were impregnated with 10 *μ*L of the respective extracts and were placed on inoculated MH agar plates that had been inoculated with the test bacteria. All the plates were incubated at 37°C for 24 h and were examined for zones of growth inhibition. The diameters of the zones of inhibition obtained after incubation were measured in millilitres for all the test organisms. The tests were performed in triplicates. Tetracycline 30 *μ*g (Oxoid Ltd, Ontario, USA) and filter paper disc soak in sterile distilled water were used as the positive and negative controls in all experiment, respectively.

### 2.6. Statistical Analysis

Descriptive statistical analysis of the antibacterial data and comparison of significant differences among the plant extracts and bacterial strains were conducted using IBM SPSS Statistics version 26 (IBM, New York, USA).

## 3. Results and Discussion

### 3.1. Chemical Compositions of *J. seravschanica* and *J. chinensis* Essential Oils

The HS-SPME-GCMS analysis of the essential oils extracted from the leaves of *J. seravschanica* and *J. chinensis* contained 37 and 36 volatiles components, respectively (Table 1). Essential oils of *J. seravschanica* and *J. chinensis* had very different chemical components make up. In total, 67 chemical compounds were detected in the essential oils of both *Juniperus* species. Most of these compounds have previously been reported to present in the essential oils of the leaves of the *Juniperus* plant from Iran. The chemical composition of the *Juniperus* leaf essential oils could be classified into five different classes of components: monoterpenes (MT), monoterpenoids (MTO), sesquiterpenes (ST), sesquiterpenoids (STO), and other, nonterpene components (NT) (Table 1). Diterpene was not detected in these essential oils.

Out of the 67 components detected in the essential oils from both of the *Juniperus* species, only 6 components, all terpenoids, were found to be present in both species. These 6 components were *α*-pinene, *β*-pinene, *γ*-elemene, sabinene, elemol, and 3-cyclohexen-1-ol (Table 1). The findings in this study were generally in agreement with the findings reported by others that *α*-pinene, *β*-pinene, sabinene, myrcene, dl-limonene, and bornyl acetate were the major components of *Juniperus* volatile oils [15]. Sela and coworkers [16] revealed two chemotypes of essential oils extracted from the berries and leaf of wild Greek Junipers (*J. excelsa* M.Bieb. (Cupressaceae) growing in the Republican of Macedonia: the *α*-pinene-type (also rich in limonene, *β*-pinene, and *β*-myrcene) and the sabinene-type (also contained *α*-pinene, *β*-myrcene, limonene, cis-thujone, terpinolene, and *α*-thujene). The *α*-pinene-type essential oil showed a moderate antibacterial activity against *S. aureus*; while, the sabinene-type essential oils were inhibitive against *E. coli*, demonstrating variable antimicrobial capacity [16] Chemical analysis conducted in this study revealed that the major chemical components in the essential oil of *J. chinensis* were *α*-pinene (27.2%), sabinene (15.2%), *α*-thujene (18.6%), and *β*-myrcene (17.9%); while, the essential oil of *J. seravschanica* was rich in *α*-pinene (33.1%) and dl-limonene (45.2%) (Table 1). The essential oils extracted from the leaf of these two species were most abundant in *α*-pinene, but only *J. chinensis* essential oil contains also sabinene. Both essential oils demonstrated comparable antimicrobial activity as given in Table 1.

### 3.2. Selective Inhibition of *J. seravschanica* and *J. chinensis* Essential Oils and Methanolic Crude Extracts against Gram-Positive and Gram-Negative Bacteria

Overall, the methanolic extracts of *J. seravschanica* showed the strongest antibacterial activity against the six bacterial strains tested (zone of inhibition = 14.3 ± 3.0 mm) compared with the methanolic extracts of *J. chinensis* (zone of inhibition = 12.6 ± 2.0 mm) and *F. bruguieri* (zone of inhibition = 12.4 ± 1.7 mm). In comparison, the essential oils of both *J. seravschanica* (zone of inhibition = 12.0 ± 2.5 mm) and *J. chinensis* (zone of inhibition = 11.9 ± 2.4 mm) demonstrated a weaker antibacterial activity based on the disc diffusion method (Table 2).

**Figure 1:**
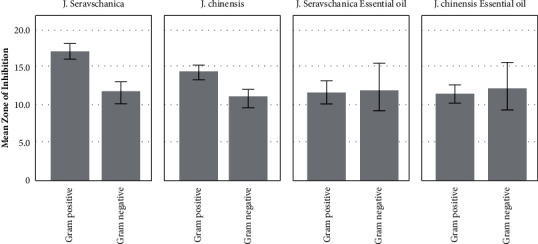
Comparison of antibacterial capacity of the methanolic extracts (100 *μ*g) of *J. seravschanica* and *J. chinensis* and essential oil of *J. seravschanica* and *J. chinensis* against Gram-positive and Gram-negative bacteria. The *p* value of the independent sample *t*-test for the methanolic extract of *J. seravschanica* and *J. chinensis* was <0.001 and for *J. seravschanica* and *J. chinensis* essential oil was >0.05.

It was noteworthy to point out that the methanolic extracts were found to have significantly higher antibacterial activity against the Gram-positive bacteria compared with the Gram-negative bacteria (*p* < 0.005); while, the essential oils of *Juniperus* did not show significant differences between Gram-positive and Gram-negative bacteria (Figure 1). A number of studies have also reported that plant extracts from *Juniperus* leaf [17] and fruit [18] could have a higher bactericidal activity against Gram-positive bacteria [17, 18]. Although another study from Oman that assess the antimicrobial activity of leaf extracts of Omani *Juniperus* excels reported only moderate antimicrobial activity of the extracts, reexamination of the data published indicates that the extracts seem to demonstrate a better activity against Gram-positive bacteria [19]. Similar scenario was also observed in extracts from other types of plants. Based on the study conducted by Rumolo and coworkers [20], *Aleurites moluccanus* (bark), *Curcuma mangga* (rhizome), and *Woodfordia floribunda* (leaf) extracts were inhibitive only against the Gram-positive bacteria (*Staphylococcus aureus* and *Enterococcus faecalis*) tested.

The observed lesser inhibitory effect against the Gram-negative bacteria was not surprising, as in general, these bacteria are more resistant than Gram-positive ones [21]. These differences may be attributed to the fact that the cell wall in Gram-positive bacteria consists of a single layer, whereas the Gram-negative bacterial cell wall is a multilayered structure and quite complex [22], with an outer membrane consisting of a hydrophilic surface rich in lipopolysaccharide molecules [23]. In addition, the periplasmic space contains enzymes that could degrade any exogenous molecules and prevent the entry of inhibitors, including antibiotic molecules [24]. Gram-positive bacteria do not possess this type of outer membrane and cell wall structure. Therefore, antibacterial substances can easily destroy the bacterial cell wall and cytoplasmic membrane and produce a leakage of the cytoplasm and its coagulation [22].

### 3.3. Antibacterial Activity of *Juniperus* Methanolic Crude Extracts against Methicillin-Resistant *Staphylococcus aureus*

Methanolic crude extract of *J. seravschanica* demonstrated better antibacterial activity against MRSA compared with the methanolic extracts of *J. chinensis* (Table 2). Methanolic crude extract of *J. seravschanica* showed high antibacterial activity against both methicillin-sensitive *S. aureus* (MSSA) and MRSA, with their inhibition zone more or less similar (Table 2). On the other hand, the methanolic extracts of *J. chinensis* and essential oils of *J. seravschanica* and *J. chinensis* showed a decreased activity against MRSA when compared with the MSSA strain tested (Table 1).

*Staphylococcus aureus*has been identified as one of the major pathogens causing community-acquired and nosocomial infections [25]. It could cause a wide range of diseases in human, including skin infections, sepsis, osteomyelitis, pneumonia, and even death. *Staphylococcus aureus* is an opportunistic pathogen that presents in the nose and skin of about 30% and 20% of healthy adults, respectively [26]. MRSA is *S. aureus* strain that has acquired multidrug resistance to beta-lactam antibiotic that is commonly used to treat bacterial infection [27]. Therefore, due to its resistance to the broad-spectrum beta-lactam antibiotics, MRSA infections are difficult to treat and pose a higher public health risk. As such, the increased effort has been focusing on searching for an alternative treatment for MRSA infection. Numerous studies have evaluated the antibacterial activity of plant compounds against MRSA. Research by Martz and coworkers [28] on the antibacterial activity of *Juniperus communis* methanolic extracts showed a satisfactory antibacterial activity against both MSSA and MRSA. Nitta and coworkers [29] evaluated the efficacy of plant extracts from 181 species of tropical and subtropical plants in inactivation of MRSA and found that the *Shorea hemsleyana* bark and *Cyphostemma bainesii* root extracts that contained stilbene derivatives demonstrated an excellent anti-MRSA activity. Other than that, essential oils of *Eucalyptus globulus*, *Thymus vulgaris, Clerodendrum serratum, Terminalia arjuna*, and *Coridothymus capitatus* showed good capability to inhibit growth of MRSA [7–11].

The better antibacterial activity of the methanolic leaf extract of *J. seravschanica* against MRSA as observed in this study requires more in-depth investigation to identify the active compounds that contribute to its inhibitory actions against MRSA.

## 4. Conclusions

In conclusion, *J. seravschanica* from Oman and *J. chinensis* from Malaysia, *Juniperus* trees of similar genus but different species as well as from different geographical locations yielded essential oils with variable chemical compositions but exert minimal differences in its antimicrobial activities. Overall, the *Juniperus* essential oils demonstrated antimicrobial activities against all the six Gram-positive and Gram-negative bacteria tested. On the other hand, different extraction methods yielded extracts with different antimicrobial capabilities. While the essential oils showed minimal selectivity in its antimicrobial activities against Gram-positive and Gram-negative bacteria, the methanolic crude extracts showed higher inhibitory activities against Gram-positive bacteria tested in this study. Also, the methanolic extract of *J. seravschanica* leaves showed a potential anti-MRSA capability. It is evident from this study that despite variation in the chemical compositions, both the *J. chinensis* and *J. seravschanica* essential oils demonstrated broad-spectrum antimicrobial activities against both Gram-positive and Gram-negative bacteria. Nevertheless, further studies are required to reveal the mechanisms of actions of the *Juniperus* essential oils against the bacteria.

## Figures and Tables

**Table 1:** Chemical composition of the essential oils extracted from the leaves of *J. chinensis* and *J. seravschanica* analysed using the HS-SPME-GCMS approach.

No.	Components	Formula	CAS	Class of components^a^	*J. chinensis*		*J. seravschanica*	
					Retention index	Contents^b^ (%)	Retention index	Contents^b^ (%)
1	Alpha-pinene	C_10_H_16_	80-56-8	MT	932	27.2	939.53	33.12
2	Sabinene	C_10_H_16_	3387-41-5	MT	948	15.2	974.9	0.05
3	Isolongifolan-8-ol	C_15_H_26_O	1139-08-8	STO	1593	3.4	1547.49	0.15
4	Beta-pinene	C_10_H_16_	127-91-3	MT	948	1.1	979.92	0.96
5	Gamma-elemene	C_15_H_24_	29873-99-2	ST	948	0.4	1306.7	0.18
6	3-Cyclohexen-1-ol	C_12_H_20_O_2_	9/4/4821	NT	1327	0.32	9.43	0.08
7	Alpha-thujene	C_10_H_16_	2/5/2867	MT	928.34	18.6	—	—
8	Beta-myrcene	C_10_H_16_	123-35-3	MT	993.82	17.82	—	—
9	Cis-caryophyllene	C_15_H_24_	6753-98-6	ST	1418.43	3.9	—	—
10	3-Carene	C_10_H_16_	554-61-0	MT	1014.74	1.8	—	—
11	Amyl ethyl ketone	C_8_H_16_O	106-68-3	NT	939.53	1.6	—	—
12	(-)-Beta-elemene	C_15_H_24_	33880-83-0	ST	897	0.8	—	—
13	4-Ethylbenzoic acid, cyclopentyl ester	C_14_H_18_O	7779-65-9	NT	1114.87	0.4	—	—
14	Cis-3-hexenol leaf alcohol	C_6_H_12_O	928-96-1	NT	1114.87	0.4	—	—
15	Cis-4-hexen-1-ol	C_6_H_12_O	821-41-0	NT	1573.08	0.33	—	—
16	4-Methoxy-2-methylbut-1-ene	C_6_H_12_O	34752-58-4	NT	1127.31	0.32	—	—
17	Amyl vinyl carbinol	C_8_H1_6_O	106-68-3	NT	1114.87	0.32	—	—
18	Alpha-humulene	C_15_H_24_	6753-98-6	ST	1452.52	0.27	—	—
19	Guaiol	C_15_H_26_O	489-86-1	NT	1082	0.2	—	—
20	1-Octen-3-ol	C_10_H_20_O	18479-51-1	NT	1652.21	0.2	—	—
21	3-Cyclohepten-1-one	C_6_H_8_O	4096-34-8	NT	1652.21	0.2	—	—
22	Hemellitol	C_9_H_12_	526-73-8	NT	1650	0.18	—	—
23	Beta-eudesmol	C_15_H_26_O	473-15-4	STO	1650	0.16	—	—
24	Cyclopropane	C_10_H_16_	68998-21-0	MT	928	0.15	—	—
25	Safrole	C_10_H_16_O	19894-97-4	MTO	1191	0.13	—	—
26	Alpha-phellandrene	C_10_H_16_	99-83-2	MT	1018.49	0.11	—	—
27	Bicyclo[3.1.0]hex-2-ene	C_10_H_16_	5/2/2867	NT	924.09	0.1	—	—
28	2-Amino-5-methylbenzoic acid	H_2_NC_6_H_3_(CH_3_)CO_2_H	2941-78-8	NT	1286.23	0.1	—	—
29	3-Octen-2-ol	C_8_H_16_O	76649-14-4	NT	1092	0.1	—	—
30	Alpha,4-Dimethylstyrene	C_10_H_12_	1195-32-0	MTO	1073	0.1	—	—
31	Bicyclo[2.2.1]heptan-7-one	C_10_H_16_O	464-48-2	MTO	1139.69	0.1	—	—
32	Bicyclo[4.1.0]hept-2-ene	C_10_H_16_	554-61-0	MT	948	0.1	—	—
33	Cis-3-hexen-1-ol	C_12_H_20_O_2_	9/4/4821	NT	1127.31	0.1	—	—
34	Cyclofenchene	C_10_H_16_	488-97-1	MT	729	0.1	—	—
35	Cyclohexane	C_12_H_18_	74742-35-1	NT	1148	0.1	—	—
36	Methyl-d3-1-dideuterio-2-propenyl ether	C_4_H_5_D_3_O	29366-08-3	NT	855.37	0.1	—	—
37	Origanene	C_10_H_16_	2/5/2867	MT	902	0.1	—	—
38	DL-Limonene	C_10_H_16_		MT	—		1037.28	45.21
39	Myrcene	C_10_H_16_	123-35-3	MT	—		993.82	8.3
40	(+)-3-Carene	C_10_H_16_	13466-78-9	MT	—		1014.74	2.81
41	Cis-calamenene	C_15_H_22_	72937-55-4	STO	—		1306.7	0.94
42	Beta-caryophyllene	C_15_H_24_	87-44-5	ST	—		1418.43	0.9
43	Alpha-copaene	C_15_H_24_	3856-25-5	ST	—		993.82	0.49
44	Bornylene	C_10_H_16_	464-17-5	MT	—		908.64	0.36
45	Delta-cadinene	C_15_H_24_	483-76-1	ST	—		1512.77	0.34
46	Caryophyllene oxide	C_15_H_24_O	1139-30-6	STO	—		1579	0.33
47	Alpha-muurolene	C_15_H_24_	1983-22-9	ST	—		974.9	0.3
48	Beta-elemene	C_15_H_24_	515-13-9	ST	—		1306.7	0.3
49	Alpha-terpinene	C_10_H_16_	99-86-5	MT	—		1018.49	0.25
50	Alpha-terpineol	C_10_H_18_O	20126-76-5	MTO	—		1640.05	0.25
51	Alpha-fenchene	C_10_H_16_	471-84-1	MT	—		950.57	0.23
52	Germacrene B	C_15_H_24_	28387-44-2	ST	—		1547.59	0.23
53	2,4(10)-Thujadiene	C_10_H_14_	36262-09-6	MTO	—		928.34	0.2
54	Alpha-campholene aldehyde	C_10_H_16_O	515-00-4	MTO	—		1513.45	0.2
55	Alpha-humulene	C_15_H_24_	6753-98-6	ST	—		948.64	0.2
56	Camphene	C_10_H_16_	79-92-5	MT	—		950.57	0.2
57	Beta-bourbonene	C_15_H_24_	5208-59-3	ST	—		908.64	0.16
58	Carvone	C_10_H_14_O	2244-16-8	OM	—		1244.05	0.15
59	Trans-pinocarveol	C_10_H_16_O	547-61-5	MTO	—		1139.69	0.15
60	Cis-(+)-carveol	C_10_H_16_O	2102-59-2	MTO	—		1219.54	0.14
61	Beta-phellandrene	C_15_H_24_	54324-03-7	ST	—		1547.59	0.13
62	Gamma-cadinene	C_15_H_24_	483-74-9	ST	—		1522.53	0.11
63	Terpinolene	C_10_H_16_	586-62-9	MT	—		1091.32	0.11
64	Beta-cubebene	C_15_H_24_	17699-14-8	ST	—		1308.8	0.1
65	Bornyl acetate	C_12_H_20_O_2_	76-49-3	NT	—		1286.23	0.1
66	Germacrene-D	C_15_H_24_	37839-63-7	ST	—		1573.08	0.1
67	Tricyclene	C_10_H_16_	508-32-7	MT	—		924.09	0.03

^a^ Monoterpenes (MT), monoterpenoids (MTO), sesquiterpenes (ST), sesquiterpenoids (STO), diterpenes (DT) and other nonterpene components (NT). ^b^The relative peak area normalization content obtained from the chromatogram.

**Table 2:** Antibacterial activity of the *J. seravschanica* and *J. chinensis* essential oils and methanolic crude extracts (100 *μ*g) of *J. seravschanica* and *J. chinensis* measured using the disc diffusion method against six bacterial strains. Each cell with different alphabet subscribes across each row was significantly different (*p* < 0.05) based on Tukey HSD post hoc analysis.

Bacteria strain	Essential oil of		Methanolic crude extracts of		Positive control (tetracycline 30 *μ*g)
	*J. seravschanica*	*J. seravschanica*	*J. seravschanica*	*J. chinensis*	
*Escherichia coli* NCTC 10418	8.1 ± 0.1^a^	9.8 ± 0.2^b^	10.9 ± 0.1^c^	12.5 ± 0.5^d^	15 ± 0.3
*Pseudomonas aeruginosa* NCTC 10662	13.2 ± 0^a^	10.7 ± 0.3^b^	10.5 ± 0.5^b^	10.7 ± 0.6^b^	15 ± 0.6
*Bacillus subtilis* ATCC 6059	12.4 ± 0.2 ^ab^	12.6 ± 0.1^b^	15.8 ± 0.3^d^	13.8 ± 0.3^c^	16 ± 0.4
*Micrococcus luteus* ATCC 10240	15.4 ± 0.2^c^	16.5 ± 0.2^d^	13.5 ± 0.5^b^	9.8 ± 0.3^a^	18 ± 0.2
*Staphylococcus aureus* NCTC 6571	13.2 ± 0.2^a^	12.2 ± 0.1^a^	17.3 ± 0.6^c^	15.3 ± 0.6^b^	17 ± 0.6
Methicillin-resistant *S. aureus* ATCC 33591	9.9 ± 0.1^a^	9.9 ± 0.2^a^	17.8 ± 0.3^c^	13.3 ± 0.6^b^	17 ± 0.6

## Data Availability

The data used to support the findings of this study are available from the corresponding author upon request.
